# Incidence of eclampsia and related complications across 10 low- and middle-resource geographical regions: Secondary analysis of a cluster randomised controlled trial

**DOI:** 10.1371/journal.pmed.1002775

**Published:** 2019-03-29

**Authors:** Nicola Vousden, Elodie Lawley, Paul T. Seed, Muchabayiwa Francis Gidiri, Shivaprasad Goudar, Jane Sandall, Lucy C. Chappell, Andrew H. Shennan, on behalf of the CRADLE Trial Collaborative Group

**Affiliations:** 1 Department of Women and Children’s Health, School of Life Course Sciences, Faculty of Life Sciences and Medicine, King’s College London, London, United Kingdom; 2 Department of Obstetrics and Gynaecology, College of Health Sciences, University of Zimbabwe, Harare, Zimbabwe; 3 Women’s and Children’s Health Research Unit, KLE Academy of Higher Education and Research, Jawaharlal Nehru Medical College, Belgaum, Karnataka, India; London School of Hygiene and Tropical Medicine, UNITED KINGDOM

## Abstract

**Background:**

In 2015, approximately 42,000 women died as a result of hypertensive disorders of pregnancy worldwide; over 99% of these deaths occurred in low- and middle-income countries. The aim of this paper is to describe the incidence and characteristics of eclampsia and related complications from hypertensive disorders of pregnancy across 10 low- and middle-income geographical regions in 8 countries, in relation to magnesium sulfate availability.

**Methods and findings:**

This is a secondary analysis of a stepped-wedge cluster randomised controlled trial undertaken in sub-Saharan Africa, India, and Haiti. This trial implemented a novel vital sign device and training package in routine maternity care with the aim of reducing a composite outcome of maternal mortality and morbidity. Institutional-level consent was obtained, and all women presenting for maternity care were eligible for inclusion. Data on eclampsia, stroke, admission to intensive care with a hypertensive disorder of pregnancy, and maternal death from a hypertensive disorder of pregnancy were prospectively collected from routine data sources and active case finding, together with data on perinatal outcomes in women with these outcomes. In 536,233 deliveries between 1 April 2016 and 30 November 2017, there were 2,692 women with eclampsia (0.5%). In total 6.9% (*n =* 186; 3.47/10,000 deliveries) of women with eclampsia died, and a further 51 died from other complications of hypertensive disorders of pregnancy (0.95/10,000). After planned adjustments, the implementation of the CRADLE intervention was not associated with any significant change in the rates of eclampsia, stroke, or maternal death or intensive care admission with a hypertensive disorder of pregnancy. Nearly 1 in 5 (17.9%) women with eclampsia, stroke, or a hypertensive disorder of pregnancy causing intensive care admission or maternal death experienced a stillbirth or neonatal death. A third of eclampsia cases (33.2%; *n =* 894) occurred in women under 20 years of age, 60.0% in women aged 20–34 years (*n =* 1,616), and 6.8% (*n =* 182) in women aged 35 years or over. Rates of eclampsia varied approximately 7-fold between sites (range 19.6/10,000 in Zambia Centre 1 to 142.0/10,000 in Sierra Leone). Over half (55.1%) of first eclamptic fits occurred in a health-care facility, with the remainder in the community. Place of first fit varied substantially between sites (from 5.9% in the central referral facility in Sierra Leone to 85% in Uganda Centre 2). On average, magnesium sulfate was available in 74.7% of facilities (range 25% in Haiti to 100% in Sierra Leone and Zimbabwe). There was no detectable association between magnesium sulfate availability and the rate of eclampsia across sites (*p =* 0.12). This analysis may have been influenced by the selection of predominantly urban and peri-urban settings, and by collection of only monthly data on availability of magnesium sulfate, and is limited by the lack of demographic data in the population of women delivering in the trial areas.

**Conclusions:**

The large variation in eclampsia and maternal and neonatal fatality from hypertensive disorders of pregnancy between countries emphasises that inequality and inequity persist in healthcare for women with hypertensive disorders of pregnancy. Alongside the growing interest in improving community detection and health education for these disorders, efforts to improve quality of care within healthcare facilities are key. Strategies to prevent eclampsia should be informed by local data.

**Trial registration:**

ISRCTN: 41244132.

## Abstract 2

Nicola vousden and colleagues reveal the highly variable care for eclampsia and other hypertensive disorders during pregnancy in low and middle income countries

## Author summary

**Why was this study done?:**

- High blood pressure in pregnancy affects nearly 1 in 7 pregnant women worldwide.
- Failure to recognise and treat this condition with antihypertensives, magnesium sulfate, and delivery of the baby can result in serious complications for the mother and baby such as eclampsia, intensive care admission, and death.
- In high-resource settings, severe complications are rare. In low-resource settings, they are known to be more common, but their frequency and impact are not as well reported.

**What did the researchers do and find?:**

- We undertook a trial introducing a novel device that measures heart rate and blood pressure into routine maternity care in 10 geographical areas in 8 countries in sub-Saharan Africa, Haiti, and India, with the aim of reducing severe pregnancy complications and death.
- In this study, we analysed all the women who experienced eclampsia or stroke or were admitted to intensive care or had a maternal death as a result of high blood pressure in pregnancy. In total, 0.5% of women experienced eclampsia (*n =* 2,692), 6.9% of these women died (*n =* 186), and 15.9% of their babies died (*n =* 397). There was substantial variation in eclampsia across different geographical regions, ranging from 0.20% to 1.42%.
- Nearly a third of eclampsia occurred in women aged under 20 years, but this proportion differed between geographical regions, ranging from 9.9% to 50.1%.
- More than half (55.1%) of women experienced their first eclamptic fit in a healthcare facility, but this proportion also differed between regions, with 6% to 85% of first eclamptic fits occurring in a central referral facility.

**What do these findings mean?:**

- This is one of the largest studies of eclampsia to date and confirms that high blood pressure in pregnancy remains an important cause of morbidity and mortality in low-resource countries.
- The majority of our geographical areas were urban or peri-urban and contained a large tertiary hospital. This means that more women with high blood pressure in pregnancy may be referred to these sites, so the rates may be higher than in the country as a whole.
- Understanding place of first eclamptic fit (in the community or in small or large tertiary facilities) may be useful in targeting interventions to reduce eclampsia, focusing either on community interventions or quality of facility care.

## Introduction

Hypertensive disorders of pregnancy cause 14% of all maternal deaths globally, approximately 42,000 each year [1,2]. Nearly all of these deaths occur in low-resource settings (99%), with death in high-income settings being very rare [3]. Hypertensive disorders of pregnancy encompass chronic hypertension, gestational hypertension (new hypertension without proteinuria), pre-eclampsia (new hypertension with proteinuria or end-organ damage after 20 weeks of gestation [4]), and eclampsia. The majority of morbidity and mortality is associated with pre-eclampsia and eclampsia.

It is estimated that the prevalence of pre-eclampsia globally is 4.6% (95% CI 2.7%–8.2%) [5]. The prevalence of eclampsia globally is reported to be 0.3% [6]. This is based on secondary analysis of a World Health Organization (WHO) multi-country survey that included 875 cases of eclampsia, collected over a short duration from only secondary or tertiary hospitals [6]. Women under 20 years of age, women with low levels of education, and women in their first pregnancy are all reported to be at higher risk [6]. Reliable data reporting the prevalence of maternal deaths related to eclampsia globally are scarce. Estimates from 16 datasets reported the case fatality rate to be 8.3% [5], whereas the WHO survey reported 32 maternal deaths, 3.7% of women with eclampsia [6]. Data from individual countries suggest that prevalence and mortality risk vary depending on region and socio-economic status [7].

Administration of magnesium sulfate more than halves the risk of eclampsia in women with pre-eclampsia [8]. It is considered an essential drug by WHO [9], but data on its availability in relation to prevalence of eclampsia are scarce [5]. Planned delivery after 36 weeks of gestation is effective at preventing maternal morbidity in women with pre-eclampsia [10]. Evidence for other interventions effective at reducing morbidity and mortality of pre-eclampsia is mixed [11], and research is generally undertaken in high-income settings, where the burden of illness is small. There is a lack of understanding around modifiable risk factors and availability of life-saving interventions, both vital in reducing the high number of deaths from this treatable cause.

The aim of this paper is to describe the incidence (per pregnancy) and characteristics of eclampsia, stroke, maternal death from hypertensive disorders of pregnancy, and intensive care unit (ICU) admission from hypertensive disorders of pregnancy across 10 geographical regions in 8 low- and middle-resource countries. The secondary aim is to describe the effect of a novel vital sign device and educational package on eclampsia, stroke, maternal death from a hypertensive disorder of pregnancy, or ICU admission with a hypertensive disorder of pregnancy.

## Methods

### Study design and setting

This is a secondary analysis of a pragmatic, stepped-wedge cluster randomised controlled trial of the introduction of the CRADLE intervention (described below) into routine maternity care in 10 sites across Zimbabwe, Zambia, Sierra Leone, Malawi, Ethiopia, Uganda, Haiti, and India over 20 months from 1 April 2016 to 30 November 2017 (ISCRTN41244132) [12,13]. The stepped-wedge design means that at the trial start all clusters commenced data collection; all clusters then crossed from control to intervention at a randomly allocated time point, at 2-monthly intervals, until all had received the intervention. The intervention effect was then determined by comparing the data points after delivery of the intervention with those in the control period. Each site comprised at least 1 secondary or tertiary hospital that provided comprehensive emergency obstetric care (central referral facility) and the main peripheral facilities that referred to the central referral facility. All secondary and tertiary hospitals were urban or peri-urban, but the geographical regions of peripheral facilities covered a range of settings, with the mean distance to the central referral facility varying from 3.3 km to 74 km. The intervention was delivered to all healthcare professionals working in the site facilities. Community healthcare providers received the intervention where they were formally involved in routine maternity care provision and supported at the district level [12].

### Intervention

The CRADLE intervention consisted of the CRADLE Vital Sign Alert, an accurate, easy-to-use device that measures maternal blood pressure and heart rate and calculates shock index. Results are displayed via a traffic light early warning system [14,15]. The devices were delivered with a one-off interactive training session of CRADLE Champions, who then provided ongoing training and support for use of the device in their clinical area. Further details of the development of the CRADLE intervention have previously been described [12,16]. This intervention was compared to routine maternity care using local management guidelines.

### Study outcomes

The primary outcome of the overall trial was a composite outcome of maternal mortality and morbidity (at least 1 of eclampsia, emergency hysterectomy, and maternal death) per 10,000 deliveries. In spite of a reduction in the primary outcome over time, this trial was unable to demonstrate an effect of the intervention. For the purpose of the analysis reported here, all women who presented to maternity care at any gestational age or up to 42 days after delivery and were diagnosed with eclampsia or experienced a complication of a hypertensive disorder of pregnancy (stroke, or being admitted to an ICU or dying as a result of a hypertensive disorder of pregnancy), between 1 April 2016 and 31 November 2017 were eligible for inclusion. The denominator was all deliveries in the trial area in the same period. Eclampsia was defined as convulsions with raised blood pressure in the absence of a known neurological cause during pregnancy or within 42 days after delivery. Other data collected included maternal age at eclamptic fit, timing of eclampsia (antenatal, including day of delivery, or postnatal), and the place of first eclamptic fit (community, peripheral facility, or central referral facility). The number of stillbirths and neonatal deaths up to 28 days was recorded for all women who had antenatal eclampsia, had a stroke, or were admitted to ICU or died as a result of hypertensive disorders of pregnancy.

Sites were described by the number of deliveries, number of ICU beds per 1,000 deliveries, and the proportion of facilities (central referral and peripheral) where magnesium sulfate was available. Availability of magnesium sulfate was recorded on a monthly basis. Details on the quantity available daily or individual-level prescriptions were not collected. Methods of data collection were discussed and optimised based on the existing resources available in each site. All data collectors were given detailed training to ensure comparability of results. Outcomes were triangulated across multiple sources (including referral registers, ward registers, patient records, local mortality and morbidity records, and active case finding) to ensure data completeness, and all outcomes were checked to avoid double counting.

### Ethics and consent

Ethical approval was granted by the Biomedical Sciences, Dentistry, Medicine and Natural and Mathematical Sciences Research Ethics Subcommittee at King’s College London (LRS-14/15-1484). This and all local ethical approvals were in place prior to the study start. Institutional-level consent on behalf of the cluster was obtained.

### Statistical methods and analysis

Statistical analyses were undertaken in Stata version 13.1. The main analysis used logistic regression with generalised estimating equations and a population-averaged model, with fixed centre effects and separate fixed linear trends in each site for changes in outcome over time [17]. Results are reported as odds ratios with 95% confidence intervals. The trial protocol stated that individual components of the primary outcome, including eclampsia, ICU admissions, and maternal deaths, and place of eclamptic fit would be analysed. However, there was no predefined analysis plan for this secondary analysis [12]. To describe the association between eclampsia and magnesium sulfate availability, the eclampsia rates for each site time period (month), and place of eclamptic fit were calculated. Eclamptic fits in the community were excluded from these analyses as magnesium sulfate was not available in the community. The analysis of the association between magnesium sulfate availability and total eclampsia by site used linear regression of the log of eclampsia rate with robust standard errors. The analysis of the association between magnesium sulfate availability and place of eclamptic fit used logistic regression with robust standard errors. Adjustments were made for time period (linear) and centre (categorical) to account for trends over time. Individual patient data were collected only for known cases.

## Results

In this cohort of 536,233 deliveries there were 2,692 cases of eclampsia over 20 months. This gives an incidence of eclampsia of 0.5%, as shown in Table 1 (50.2/10,000 deliveries). In total, 6.9% (*n =* 186; 3.47/10,000 deliveries) of women with eclampsia died (sepsis [*n =* 4], stroke [*n =* 4], haemorrhage [*n =* 18], hypertensive disorders of pregnancy [*n =* 150], and other causes [*n =* 10]), and a further 51 women died from other complications of hypertensive disorders of pregnancy without having had eclampsia (0.95/10,000). Eight of the 10 sites had capacity for ICU admission, although availability of beds varied between sites (S1 Table). In total, 1,322 women were admitted to ICU with hypertensive disorders of pregnancy, 27.8% of these with eclampsia (*n =* 367) and 72.2% (*n =* 955) with other complications of hypertensive disorders of pregnancy. After planned adjustments for clustering and time trends in each site, the implementation of the CRADLE intervention was not associated with any significant change in the rates of eclampsia, stroke, maternal death from a hypertensive disorder of pregnancy, or ICU admission with a hypertensive disorder of pregnancy.

**Table 1:** Rates of eclampsia, stroke, and maternal death and intensive care unit admission as a result of hypertensive disorders of pregnancy, and effect of the CRADLE intervention on outcomes.

Outcome	Rate/10,000 deliveries; *n/N*			Odds ratio (95% CI)	
	Overall	Pre-intervention	Post-intervention	Unadjusted comparison	Adjusted comparison^1^
**All eclampsia**	50.20 2,692/536,233	53.15 1,314/247,238	47.77 1,378/288,995	0.90 (0.83–0.97)	1.30 (0.82–2.05)
**All maternal death from hypertensive disorder of pregnancy**^**2**^	4.42 237/536,233	3.84 95/247,238	4.91 142/288,995	1.28 (0.99–1.66)	0.84 (0.47–1.51)
Maternal death with eclampsia	3.47 186/536,233	2.87 71/247,238	3.98 115/288,995	1.39 (1.03–1.86)	0.79 (0.31–2.02)
Maternal death without eclampsia	0.95 51/536,233	0.97 24/247,238	0.93 27/288,995	0.96 (0.56–1.67)	1.06 (0.38–2.93)
**ICU admission from hypertensive disorder of pregnancy**^**2**^	24.65 1,322/536,223	26.57 657/247,238	23.01 665/288,995	0.87 (0.78–0.96)	0.85 (0.72–1.02)
ICU admission with eclampsia	6.8 367/536,233	9.18 227/247,238	4.84 140/288,995	0.53 (0.43–0.65)	0.71 (0.38–1.33)
ICU admission without eclampsia	17.81 955/536,233	17.39 430/247,238	18.17 525/288,995	1.04 (0.92–1.19)	0.92 (0.87–0.96)
**Stroke**	0.62 33/536,233	0.85 21/247,238	0.42 12/288,995	0.49 (0.24–0.99)	2.08 (0.58–7.55)

^1^Adjusted for time period and centre to account for trends over time.

^2^All maternal death and ICU admission from hypertensive disorder of pregnancy include all cases resulting from hypertensive disorder of pregnancy, including those with and without eclampsia. Both groups are then subdivided into those with eclampsia and those secondary to other complications of hypertensive disorders of pregnancy without having eclampsia.

ICU, intensive care unit.

**Fig 1:**
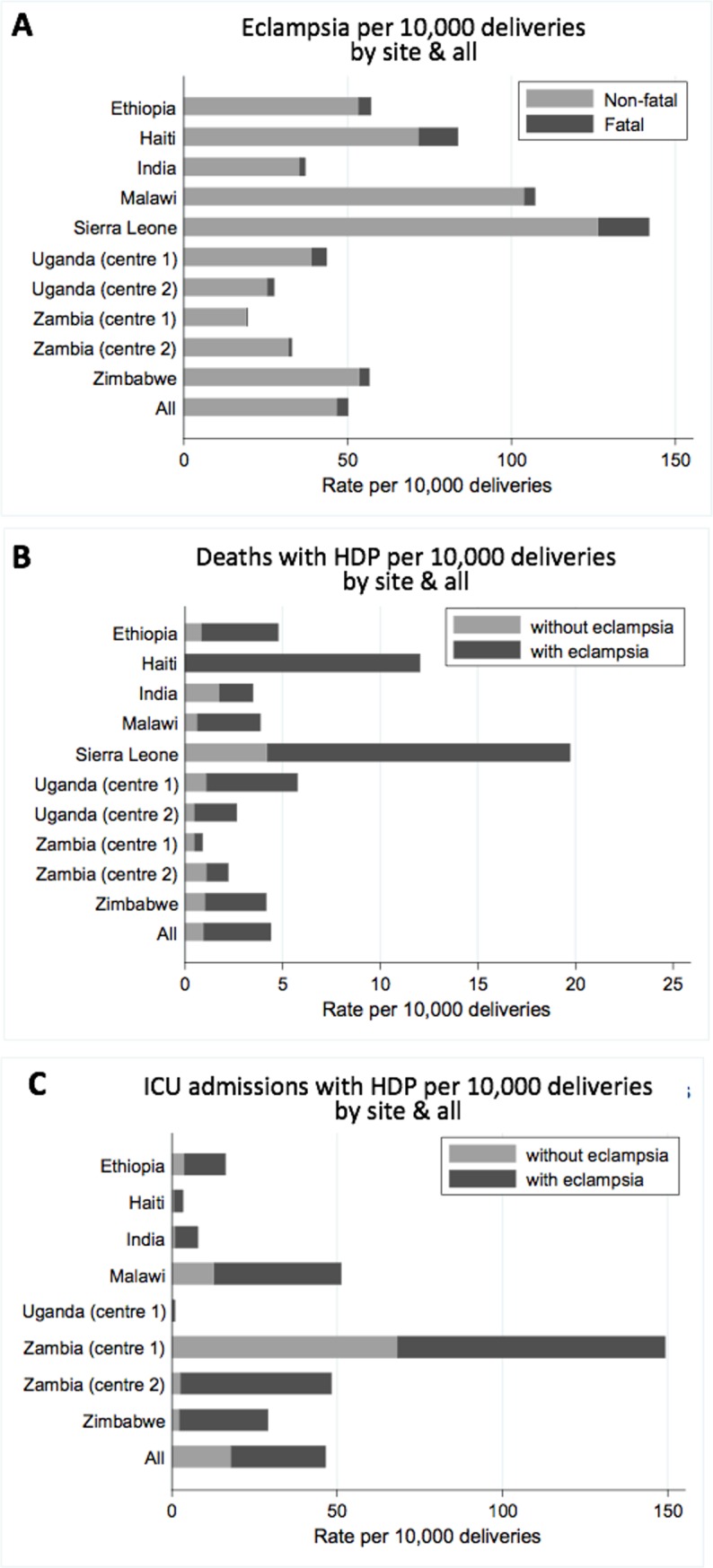
Rates of eclampsia and maternal deaths and intensive care unit (ICU) admissions from hypertensive disorders of pregnancy (HDP) by site. Data shown for eclampsia (A), maternal deaths from HDP (B), and ICU admissions from HDP (C). ICU admissions shown for sites with ICU available. Corresponding numbers are given in S2 and S3 Tables.

Rates of eclampsia varied between sites from 19.6 per 10,000 deliveries in Zambia Centre 1 (Lusaka) to 142.0 per 10,000 deliveries in Sierra Leone (Fig 1; S2 Table). When comparing the effect of the intervention across individual sites, further consideration is required as these are non-randomised data and are vulnerable to external influences such as seasonal trends. After planned adjustment, there was a significant reduction in eclampsia in Haiti and Zambia Centre 1, a significant increase in Malawi and Uganda Centre 2, and no significant changes in other sites (S2 Table). The rate of maternal death from eclampsia in each site largely reflected the incidence of eclampsia (from 0.4 per 10,000 deliveries in Zambia Centre 1 to 15.5 per 10,000 deliveries in Sierra Leone; S3 Table). The range of case fatality rate for women with eclampsia was from 2.1% (5/242) in Zambia Centre 1 to 14.4% (18/125) in Haiti. Only 33 in the cohort of 536,233 women were diagnosed with a stroke. ICU admission from hypertensive disorders of pregnancy also varied between sites as shown in Fig 1 (S3 Table).

**Fig 2:**
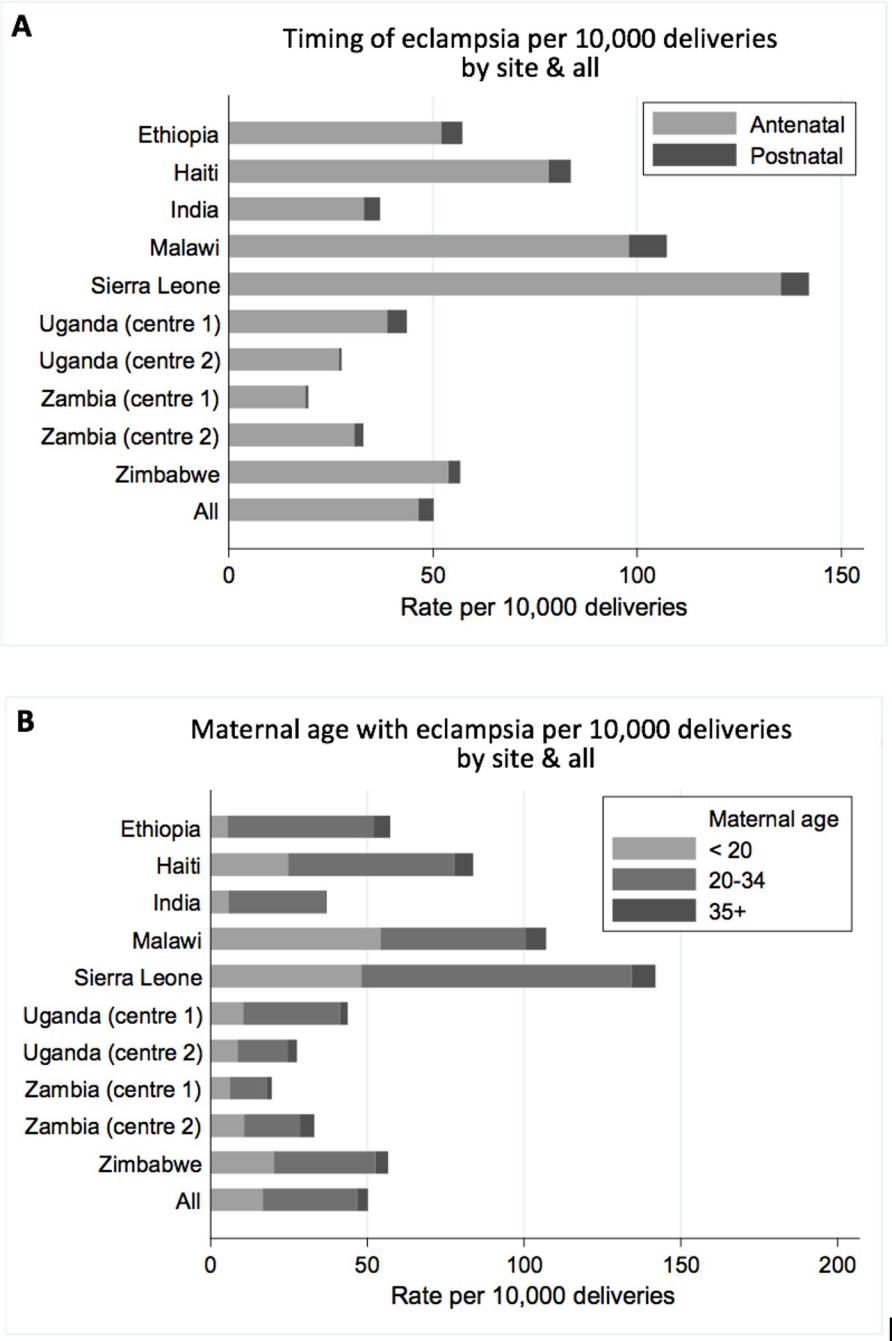
Characteristics of eclampsia by timing and maternal age by site. Data shown by timing of eclampsia (A) and maternal age (B). Corresponding numbers are given in S4 Table.

Across all sites, 92.7% (*n =* 2,495) of eclampsia cases occurred in the antenatal period and 7.3% (*n =* 197) in the postnatal period. The proportion of eclampsia cases occurring in the antenatal period was similar across sites (range 88.9% in Uganda Centre 1 to 98.2% in Uganda Centre 2) (Fig 2; S4 Table). Approximately a third of eclampsia cases (33.2%; *n =* 894) occurred in women aged under 20 years. This proportion varied between sites from 10% in Ethiopia to 51% in Malawi (Fig 2; S4 Table). The majority of eclampsia cases occurred in women aged 20–34 years (60.0%; *n =* 1,616); 6.8% (*n =* 182) occurred in women aged 35 years or over.

In total, there were 10 central referral facilities and 268 peripheral facilities. Nearly half of all first eclamptic fits occurred in the community (44.9%; range 30.8% in Malawi to 66.0% in Freetown; Table 2), with 31.2% occurring for the first time in central referral facilities (range 5.9% in Sierra Leone to 85.0% in Uganda Centre 2) and 23.9% in peripheral facilities (range 4.7% in India to 33.0% in Ethiopia). On average, magnesium sulfate was available in 74.7% of facilities (range 25% in Haiti to 100% in Sierra Leone and Zimbabwe). Availability of magnesium sulfate did not significantly change during the trial period. There was no significant association between the overall availability of magnesium sulfate in central referral and peripheral facilities and the proportion of eclampsia cases that occurred in each (central referral facilities: *p =* 0.42; peripheral facilities: *p =* 0.13; Table 2). There was also no detectable association between the proportion of facilities with magnesium sulfate available across the sites and the rate of eclampsia in each site (*p =* 0.12). Of the 1,210 women who had their first eclamptic fit in the community, 7.5% (91/1,210) died; of the 1,482 who had their first eclamptic fit in a facility, 6.4% (95/1,482) died.

**Table 2:** Place of eclamptic fit and proportion of facilities with magnesium sulfate available on average over trial duration.

Site	Total *n*	*n* (percent of eclampsia cases)			Mean magnesium sulfate availability over trial duration (%)
		Central referral facility	Peripheral facility	Community	
**All sites**	**2,692**	**839 (31.2%)**	**643 (23.9%)**	**1,210 (44.9%)**	**74.7%**
Ethiopia	203	57 (28.1%)	67 (33.0%)	79 (38.9%)	87.4%
Haiti	125	52 (41.6%)	16 (12.8%)	57 (45.6%)	25.0%
India	85	27 (31.8%)	4 (4.7%)	54 (63.5%)	95.4%
Malawi	666	242 (36.3%)	219 (32.9%)	205 (30.8%)	100.0%
Sierra Leone	338	20 (5.9%)	95 (28.1%)	223 (66.0%)	41.4%
Uganda Centre 1	559	161 (28.8%)	125 (22.4%)	273 (48.8%)	61.5%
Uganda Centre 2	167	142 (85.0%)	17 (10.2%)	8 (4.8%)	78.6%
Zambia Centre 1	242	34 (14.0%)	61 (25.2%)	147 (60.7%)	79.8%
Zambia Centre 2	89	38 (42.7%)	13 (14.6%)	38 (42.7%)	77.4%
Zimbabwe	218	66 (30.3%)	26 (11.9%)	126 (57.8%)	100.0%

In the group of 3,493 women with antenatal eclampsia (*n =* 2,495), stroke, or hypertensive disorders of pregnancy causing ICU admission or maternal death (*n =* 998), the rate of stillbirth or neonatal mortality was very high (17.9%; *n =* 625). The rate of stillbirth or neonatal mortality was higher in women with hypertensive disorders of pregnancy (i.e., resulting in stroke, ICU admission, or maternal death) without eclampsia than in women with eclampsia (stillbirth or neonatal death: 22.8% [*n =* 228] in women with hypertensive disorders of pregnancy without eclampsia and 15.9% [*n =* 397] in women with eclampsia; Table 3). Overall rate of stillbirth or neonatal mortality in women with eclampsia varied between sites from 4.1% in Malawi to 23.1% in Uganda Centre 1 (S5 Table).

**Table 3:** Perinatal outcomes for mothers with antenatal eclampsia, stroke, or hypertensive disorders of pregnancy causing death or intensive care unit (ICU) admission.

Perinatal outcomes	Overall *n/N* (%)
**All stillbirth and neonatal death in women with eclampsia, stroke or ICU admission or maternal death from hypertensive disorders of pregnancy**	625/3,493 (17.9%)
**All stillbirth and neonatal death in women with antenatal eclampsia**	397/2,495 (15.9%)
Pregnancies ending in stillbirth	322/2,495 (12.9%)
Neonatal death	75/2,495 (3.0%)
**All stillbirth and neonatal death in women with hypertensive disorders of pregnancy causing stroke, ICU admission or death without eclampsia**	228/998 (22.8%)
Pregnancies ending in stillbirth	197/998 (19.7%)
Neonatal death	31/998 (3.1%)

Excludes 12 with missing delivery information and 45 that went home after eclampsia without delivery and were not followed up.

## Discussion

Overall, we report that 0.5% of women in our sites experienced eclampsia, 57.2% of women with eclampsia were admitted to ICU, and 6.9% died. Our individual site analysis shows large variation both in the rate of eclampsia and in the rates of maternal death and ICU admission from hypertensive disorders of pregnancy. Stroke was a rare outcome in all of our sites. The majority of eclampsia cases across all sites first occurred in the community (44.9%), in the antenatal period (92.6%), and in women aged 20–34 years (60.0%). Overall, the implementation of the CRADLE intervention was not associated with any significant change in the rate of eclampsia, stroke, or maternal death or ICU admission with hypertensive disorders of pregnancy, but the effect in individual sites varied.

To our knowledge, this is the largest prospectively collected dataset on women with eclampsia. The strengths of these data are the rigorous method of prospective data collection, verified from multiple sources, and inclusion of multiple countries and settings. The majority of existing data have focused on eclampsia presenting to secondary or tertiary hospitals [6]. The data presented here improve the accuracy of incidence estimates by including cases across the health system, including cases from primary healthcare facilities and community cases.

Although the geographical settings of this study varied, it is a limitation of this study that the majority of sites were urban or peri-urban. These settings were selected as a substantial proportion of births occur in them. This approach, in addition to the inclusion of the national referral hospital in many of our sites, means the incidence of complications from hypertensive disorders of pregnancy in our sites may be higher than country-wide levels. Due to the size of the study, it was not feasible to collect demographic data in the population of women who delivered in the trial area. Therefore, the proportion of eclampsia cases in different age groups and perinatal outcomes cannot be presented at the population level. Effects of the intervention on perinatal outcomes or by age or place of eclampsia are therefore not presented. The effect of the intervention in individual sites needs further consideration as these are non-randomised analyses. The numbers of cases of eclampsia and hypertensive disorders of pregnancy were based on the data reported by attending clinicians in the health facilities; it was not feasible to undertake additional searching in all sites to identify cases not reported. However, the inclusion of maternal deaths and ICU admissions only from hypertensive disorders of pregnancy means that misdiagnosis is less likely. Whilst it is a strength that this paper reports magnesium sulfate availability, this variable was collected on a monthly basis at the level of the facility. As daily fluctuations in the quantity available or the number of doses prescribed remain unknown, it is possible that supply may not have been adequate to meet demand.

In the post–Millennium Development Goal era, the focus of global health is on not just reducing mortality but also reducing morbidity [18]. Yet, in this study, the large variation in fatality from eclampsia between countries emphasises that both inequality and inequity in management of hypertensive disorders of pregnancy persist. It has been previously reported that organ dysfunction is up to 60 times more frequent in women with eclampsia compared to women without eclampsia [6]. Therefore, the very high rates of maternal death in some countries compared to previously reported estimates [6,7] highlight that the true burden of disease in these countries may be even greater than previously recognised and that hypertensive disorders of pregnancy should remain firmly on the global agenda.

This study showed no effect of the CRADLE intervention on eclampsia, stroke, or maternal death or ICU admission from hypertensive disorders of pregnancy. It is possible that the intervention increased detection but without the capacity to improve management. The primary purpose of the study was to collect accurate incidence data, and therefore detailed case information on clinical management was not routinely collected, given the resource constraints of the trial. It is challenging therefore to draw conclusions on differences in management that may also have contributed to variations in the rate of eclampsia and resulting morbidity seen. However, it is evident that Zambia Centre 1 (Lusaka) had the lowest rate of eclampsia and case fatality, and admitted substantially more patients to ICU than any other site. This was possible as they have a specialist unit specifically for women with hypertensive disorders of pregnancy that provides continuous monitoring and close observation by trained staff. In comparison, the site in Freetown in Sierra Leone, which had the highest rate of eclampsia and case fatality, has no higher-level care available. The availability of monitoring to rapidly detect deteriorations—and initiate treatment such as antihypertensives, magnesium sulfate, and timely delivery—is likely to be important. This idea is in keeping with reports that the largest reductions in maternal mortality from hypertensive disorders of pregnancy in England and Wales were achieved with improved surveillance, diagnosis, and timely delivery, with further benefit from fluid-restriction management protocols and increased use of anticonvulsant therapies in more recent decades [19].

In this study, nearly a third of eclampsia cases occurred in women aged under 20 years. This proportion varied greatly between sites, with the Malawi site reporting that half of eclampsia cases occurred in women aged under 20 years. Other studies have reported rates of 26% [6] to 55% [20]. Whilst this study did not collect demographic data in all deliveries, nationwide demographic data show that 15% of births in Malawi in 2015–2016 occurred in women aged under 20 years [21]. Existing literature suggests that teenage pregnant women are at greater risk of eclampsia [22], and their care should be prioritised in clinical practice. Interventions aiming to overcome the complex socio-cultural needs of this group to improve access to healthcare and prevent eclampsia warrant further research.

This study also presents novel data on the place of eclamptic fit, previously only reported in smaller cohort studies, where 74.5% (*n =* 142) were reported to occur before hospital admission in Latin America [20]. Our data demonstrate that over half of women experience their first eclamptic fit in a healthcare facility, despite the relatively good availability of magnesium sulfate in these settings. However, the proportion of eclampsia cases that first occurred in healthcare facilities compared to the community varied substantially between sites. This suggests that the most appropriate interventions and strategies to reduce eclampsia should be informed by local incidence data. For example, in Sierra Leone, Zambia Centre 1, and India, where a high proportion of eclampsia cases occurred in the community, interventions aiming to improve community monitoring and overcome barriers to accessing care, including health education [23], may be the most appropriate use of resources. In contrast, in Uganda Centre 2 and Haiti, targeting the quality of care within facilities may be a more effective strategy for preventing eclampsia. Therefore, when vital actions such as treating severe hypertension with magnesium sulfate to prevent eclampsia [8] and timely delivery of the baby [10] are recommended, national and international policy makers may also recommend collection of regional data to identify how these interventions should be delivered to achieve the greatest benefit locally, thus maximising their impact and identifying the most appropriate use of resources.

In conclusion, this analysis provides accurate contemporaneous estimates of incidence of eclampsia and hypertensive disorders of pregnancy from the largest known prospective dataset across 8 low- and middle-resource settings. These data highlight that mortality (for the woman and baby) from eclampsia remains high, and higher-risk groups exist that should be prioritised in research and policy. Use of magnesium sulfate to prevent eclampsia and timely delivery after diagnosis remain important strategies to reduce maternal and perinatal mortality from hypertensive disorders of pregnancy at the facility level, but interventions should also be targeted to meet the needs of specific regions.

## Supporting information

S1 Checklist STROBE statement. (DOC)

S1 Table Characteristics of sites. (DOCX)

S2 Table Eclampsia by site and by intervention. (DOCX)

S3 Table Maternal deaths from hypertensive disorders of pregnancy, ICU admissions with hypertensive disorders of pregnancy, and stroke by site. (DOCX)

S4 Table Characteristics by site. (DOCX)

S5 Table Perinatal outcomes for mothers with hypertensive disorders of pregnancy by site. (DOCX)

S6 Table Trial facilities per site. (DOCX)

## Abbreviations

ICU: intensive care unit
WHO: World Health Organization
