# Supplementary material for: Incidence of eclampsia and related complications across 10 low- and middle-resource geographical regions: Secondary analysis of a cluster randomised controlled trial
Source: PLoS Med. 2019 Mar 29;16(3):e1002775. doi: 10.1371/journal.pmed.1002775 (PMC6440614; doi:10.1371/journal.pmed.1002775)
Supplement: S1 Checklist — (DOC) [file pmed.1002775.s001.doc]

**S1 Checklist: STROBE Statement**

|  | Item No | Recommendation | Section and Paragraph number | Text Excerpt |
| --- | --- | --- | --- | --- |
| **Title and abstract** | 1 | (*a*) Indicate the study’s design with a commonly used term in the title or the abstract | Abstract - Title | “Incidence of eclampsia and related complications across ten low and middle-resource geographical regions: secondary analysis of a cluster randomised controlled trial.” |
|  |  | (*b*) Provide in the abstract an informative and balanced summary of what was done and what was found | Abstract – Methods and Results | “This is a secondary analysis of a stepped-wedge cluster randomised-controlled trial undertaken in sub-Saharan Africa, India and Haiti (ISRCTN41244132). This trial implemented a novel vital sign device and training package into routine maternity care with the aim of reducing a composite outcome of maternal mortality and morbidity. Institutional-level consent was obtained and all women presenting for maternity care were eligible for inclusion. Eclampsia, stroke, admission to intensive care and maternal death from hypertensive disorders of pregnancy data were prospectively collected from routine data sources and active case finding, together with perinatal outcomes in women with these outcomes. Between 01st April 2016 and 30th November 2017 in 536,233 deliveries there were 2692 women with eclampsia (0.5%). In total 6.9% (n=186; 3.47/10,000 deliveries) of women with eclampsia died and a further 51 died from other complications of hypertensive disorders of pregnancy (0.95/10,000). Nearly one in five (17.9%) women with eclampsia, stroke and hypertensive disorders of pregnancy causing intensive care admission or maternal death, experienced a stillbirth or neonatal death. A third of eclampsia (33.2%; n=894) occurred in women under 20 years of age, 60.0% in women aged 20-34 years (n=1616) and 6.8% (n=182) in women aged 35 or over. Rates of eclampsia varied approximately seven-fold between sites (range 19.6/10,000 in Zambia Centre 1 to 142.0/10,000 in Sierra Leone). Over half (55.1%) of first eclamptic fits occurred in a health care facility, with the remainder in community. Place of first fit varied substantially between sites (from 5.9% in the central referral facility in Sierra Leone to 85% in Uganda Centre 2). On average, magnesium sulfate was available in 74.7% of facilities (range 25% in Haiti to 100% in Sierra Leone and Zimbabwe). There was no detectable association between magnesium sulfate availability and the rates of eclampsia in each site (p=0.12). This analysis may be influenced by the selection of predominantly urban and peri-urban settings including tertiary referral hospitals, monthly data on availability of magnesium sulfate, and is limited by the lack of demographic data in the denominator group. ” |
| Introduction | | |  |  |
| Background/rationale | 2 | Explain the scientific background and rationale for the investigation being reported | Introduction paragraph 1 & 2 | Literature reviewed as per introduction |
| Objectives | 3 | State specific objectives, including any prespecified hypotheses | Introduction paragraph 5 | “The aim of this paper is to describe the incidence (per pregnancy) and characteristics of eclampsia and maternal death from severe hypertensive disorders of pregnancy across ten geographical regions in eight low and middle-resource countries. The secondary aim is to describe the effect of novel vital sign device and educational package on eclampsia and its complications.” |
| Methods | | |  |  |
| Study design | 4 | Present key elements of study design early in the paper | Method paragraph 1 | “This is a secondary analysis of a pragmatic, stepped-wedge cluster randomised controlled trial of the introduction of the CRADLE intervention (described below)” |
| Setting | 5 | Describe the setting, locations, and relevant dates, including periods of recruitment, exposure, follow-up, and data collection | Method paragraph 1 ‘Study design’ | “into routine maternity care in ten sites across Zimbabwe, Zambia, Sierra Leone, Malawi, Ethiopia, Uganda, Haiti and India over 20 months from 1^st^ April 2016 to 30^th^ November 2017 (ISCRTN: 41244132). Each site comprised at least one secondary or tertiary health facility that provided comprehensive emergency obstetric care with the main peripheral facilities that refer to the central hospital. All secondary or tertiary hospitals were urban or peri-urban, but the geographical regions of peripheral facilities covered a range of settings with the mean distance varying from 3.3km to 74km.” |
| Participants | 6 | (*a*) *Cohort study*—Give the eligibility criteria, and the sources and methods of selection of participants. Describe methods of follow-up | Method paragraph 3 ‘Study outcomes’ | “For the purpose of the analysis reported here, all women that presented to maternity care at any gestation or up to 42 days after delivery and were diagnosed with eclampsia or stroke, or were admitted to intensive care (ICU) or died as a result of hypertensive disorders of pregnancy, between 01/04/2016 and 31/11/2017 were eligible for inclusion. The denominator was all deliveries in the trial area in the same period.” |
|  |  | (*b*) *Cohort study*—For matched studies, give matching criteria and number of exposed and unexposed  *Case-control study*—For matched studies, give matching criteria and the number of controls per case | NA | NA |
| Variables | 7 | Clearly define all outcomes, exposures, predictors, potential confounders, and effect modifiers. Give diagnostic criteria, if applicable | Method paragraph 3&4  ‘Study outcomes’ | As above + “Eclampsia was defined as convulsions with raised blood pressure in the absence of a known neurological cause during pregnancy or within 42 days of delivery. Other data collected included maternal age at eclamptic fit, timing of eclampsia (antenatal including day of delivery or postnatal) and the place of first eclamptic fit (community, peripheral facility or central referral facility). The number of stillbirths and neonatal deaths up to 28 days were recorded in all women that had antenatal eclampsia, and all women that had a stroke, were admitted to ICU or died as a result of hypertensive disorders of pregnancy. Sites were described by the number of deliveries, number of ICU beds per 1000 deliveries and the proportion of facilities (central or peripheral) where magnesium sulfate was available.” |
| Data sources/ measurement | 8* | For each variable of interest, give sources of data and details of methods of assessment (measurement). Describe comparability of assessment methods if there is more than one group | Method paragraph 4  ‘Study outcomes’ | “Availability of magnesium sulfate was recorded on a monthly basis. Details on the quantity available daily or individual level prescriptions were not collected*.* Methods of data collection were discussed and optimised based on the existing resources available in each site. All data collectors were given detailed training to ensure comparability of results. Outcomes were triangulated across multiple sources (including referral registers, ward registers, patient records, local mortality and morbidity records, active case finding) to ensure data completeness and all outcomes checked to avoid double counting.” |
| Bias | 9 | Describe any efforts to address potential sources of bias | Method paragraph 6 ‘Statistical Analysis’ | “Adjustments were made for period (linear) and centre (categorical) to account for trends over time.” |
| Study size | 10 | Explain how the study size was arrived at | NA | Secondary analysis of RCT |
| Quantitative variables | 11 | Explain how quantitative variables were handled in the analyses. If applicable, describe which groupings were chosen and why | Method paragraph 6 ‘Statistical Analysis’ | “To describe the association between eclampsia and magnesium sulfate availability, eclampsia rates for each centre, time period (month) and place of eclamptic fit were calculated. Community fits were excluded from these analyses as magnesium sulfate was not available in the community. The association between magnesium sulfate availability and total eclampsia by site used linear regression with the log of eclampsia rate with robust standard errors. The association between magnesium sulfate availability and place of fit used logistic regression with robust standard errors. Adjustments were made for period (linear) and centre (categorical) to account for trends over time. Individual patient data were collected only for known cases.” |
| Statistical methods | 12 | (*a*) Describe all statistical methods, including those used to control for confounding | Method paragraph 6 ‘Statistical Analysis’ | As above. |
|  |  | (*b*) Describe any methods used to examine subgroups and interactions | Method paragraph 6 ‘Statistical Analysis’ | As above |
|  |  | (*c*) Explain how missing data were addressed | Method paragraph 6 ‘Statistical Analysis’ and footnote of Table | “Individual patient data were collected only for known cases.”  “Perinatal outcome data excludes 12 with missing delivery information and 45 that went home after eclampsia without delivery.” |
|  |  | (*d*) *Cohort study*—If applicable, explain how loss to follow-up was addressed | NA | NA |
|  |  | (*e*) Describe any sensitivity analyses | NA | NA |

| Results | | |  |  |
| --- | --- | --- | --- | --- |
| Participants | 13* | (a) Report numbers of individuals at each stage of study—eg numbers potentially eligible, examined for eligibility, confirmed eligible, included in the study, completing follow-up, and analysed |  | Since this was a cluster randomised controlled trial and cluster level consent was gained, as opposed to individual consent, all women presenting to maternity care were eligible for inclusion with no exclusion criteria or possibility of non-participation. |
|  |  | (b) Give reasons for non-participation at each stage |  |  |
|  |  | (c) Consider use of a flow diagram |  |  |
| Descriptive data | 14* | (a) Give characteristics of study participants (eg demographic, clinical, social) and information on exposures and potential confounders | S1 Table |  |
|  |  | (b) Indicate number of participants with missing data for each variable of interest | Table Footnote | “Perinatal outcome data excludes 12 with missing delivery information and 45 that went home after eclampsia without delivery.” |
|  |  | (c) *Cohort study*—Summarise follow-up time (eg, average and total amount) | NA |  |
| Outcome data | 15* | *Cohort study*—Report numbers of outcome events or summary measures over time | Results paragraph 1 | In this cohort of 526,233 deliveries there were 2692 cases of eclampsia over 20-months. |
| Main results | 16 | (*a*) Give unadjusted estimates and, if applicable, confounder-adjusted estimates and their precision (eg, 95% confidence interval). Make clear which confounders were adjusted for and why they were included | S2 Table | Not applicable for main aim of paper but presented for secondary aim in Supplementary table S2. “Both were adjusted for period and centre to account for underlying trends over time.” |
|  |  | (*b*) Report category boundaries when continuous variables were categorized |  | NA |
|  |  | (*c*) If relevant, consider translating estimates of relative risk into absolute risk for a meaningful time period |  | NA |
| Other analyses | 17 | Report other analyses done—eg analyses of subgroups and interactions, and sensitivity analyses |  | NA |
| Discussion | | |  |  |
| Key results | 18 | Summarise key results with reference to study objectives | Discussion paragraph 1 | “Overall, we have reported that 0.5% of women in our sites experienced eclampsia, 57.2% of women with eclampsia are admitted to ICU and 6.9% died. Our individual site analysis has shown large variation both in the rates of eclampsia, but also in the rates of maternal death and ICU admission from hypertensive disorders of pregnancy. Stroke was a rare outcome in all of our sites. The majority of eclampsia across all sites first occurs in the community (44.9%), in the antenatal period (92.6%) and in women aged 20-34 (60.0%). Overall, the implementation of the CRADLE intervention was not associated with any significant change in the rates of eclampsia, stroke, maternal death or ICU admission with hypertensive disorders of pregnancy but the effect in individual sites varied.” |
| Limitations | 19 | Discuss limitations of the study, taking into account sources of potential bias or imprecision. Discuss both direction and magnitude of any potential bias | Discussion paragraph 3 | “Although the geographical settings varied, it is a limitation of this study that the majority of sites were urban or peri-urban. These were selected as a substantial proportion of births occur here. This, in addition to the inclusion of the national referral hospital in many of our sites, means the incidence of complications from hypertensive disorders of pregnancy may be higher than country wide levels. Due to the size of the study it was not feasible to collect demographic data in the denominator group, therefore the proportion of eclampsia in different age groups and perinatal outcomes cannot be presented at the population level. Comparisons by intervention on perinatal outcomes or age or place of eclampsia are therefore not presented. The effect of the intervention in individual sites needs further consideration as this is non-randomised analyses. The number of eclampsia and hypertensive disorders of pregnancy were based on the data reported by attending clinicians in the selected hospitals; it was not feasible to undertake additional searching in all sites to identify cases not reported; however, the inclusion of only maternal death and ICU admission from hypertensive disorders of pregnancy mean that mis-diagnosis is less likely. Whilst it is a strength that this paper reports magnesium sulfate availability, this was collected on a monthly basis at the level of the facility. As daily fluctuations in the quantity available, or the number of doses prescribed remains unknown, it is possible that supply may not have been adequate to meet demand.” |
| Interpretation | 20 | Give a cautious overall interpretation of results considering objectives, limitations, multiplicity of analyses, results from similar studies, and other relevant evidence | Discussion paragraph 8 | “In conclusion, this analysis provides accurate contemporaneous estimates of incidence of eclampsia and severe hypertensive disorders of pregnancy from the largest known prospective dataset across eight low and middle-resource settings. These data highlight that mortality (for the woman and baby) from eclampsia remains high and higher-risk groups exist that should be prioritized in research and policy. Use of magnesium sulfate to prevent eclampsia and timely delivery after diagnosis remain important strategies to reduce maternal and perinatal mortality from hypertensive disorders of pregnancy at the facility level but interventions should also be targeted to meet the need of the region.” |
| Generalisability | 21 | Discuss the generalisability (external validity) of the study results | Discussion paragraph 3 | “Although the geographical settings varied, it is a limitation of this study that the majority of sites were urban or peri-urban. These were selected as a substantial proportion of births occur here. This, in addition to the inclusion of the national referral hospital in many of our sites, means the incidence of complications from hypertensive disorders of pregnancy may be higher than country wide levels. Due to the size of the study it was not feasible to collect demographic data in the denominator group, therefore the proportion of eclampsia in different age groups and perinatal outcomes cannot be presented at the population level.” |
| Other information | | |  |  |
| Funding | 22 | Give the source of funding and the role of the funders for the present study and, if applicable, for the original study on which the present article is based | Funding Statement | The CRADLE Trial was funded by the Medical Research Council, Department of Biotechnology India and Department of International Development joint fund. The funders had no input into the conduct or output of this paper. |

*Give information separately for cases and controls in case-control studies and, if applicable, for exposed and unexposed groups in cohort and cross-sectional studies.
