# Supplementary material for: Incidence of eclampsia and related complications across 10 low- and middle-resource geographical regions: Secondary analysis of a cluster randomised controlled trial
Source: PLoS Med. 2019 Mar 29;16(3):e1002775. doi: 10.1371/journal.pmed.1002775 (PMC6440614; doi:10.1371/journal.pmed.1002775)
Supplement: S1 Table — (DOCX) [file pmed.1002775.s002.docx]

**S1 Table: Characteristics of Sites**

| **Site** | **Number of primary level care facilities:** | **Number of secondary level care facilities:** | **Number of tertiary level care facilities:** | **Adult Intensive Care Unit beds** | **MgSO4 availability** | **Distance from peripheral facilities to nearest tertiary facility** |
| --- | --- | --- | --- | --- | --- | --- |
|  | n/1000 deliveries | n/1000 deliveries | n/1000 deliveries | n per 1,000 deliveries^1^ | mean availability % over trial duration | Mean (km) (SD) |
| **Ethiopia** | 9.7 | 0.6 | 0.6 | 7.4 | 87.4% | 4.3 (2.7) |
| **Haiti** | 11.7 | 1.5 | 4.4 | 0 | 25.0% | 14.2(5.4) |
| **India** | 55.9 | 18.6 | 0 | 24.5 | 41.4% | 74 (16.9) |
| **Malawi** | 1.0 | 2.6 | 0.3 | 5.3 | 95.4% | 68.3 (35.2) |
| **Sierra Leone** | 9.4 | 0.9 | 0.9 | 0 | 100.0% | 7.5 (3.7) |
| **Uganda Centre 2** | 13.6 | 0.3 | 0 | 3.3 | 79.8% | 8.7 (4.3) |
| **Uganda Centre 1** | 1.3 | 0.6 | 0.2 | 0 | 78.6% | 19.6 (12.4) |
| **Zambia Centre 1** | 4.1 | 0.2 | 0.2 | 2.7 | 61.5% | 3.3 (1.3) |
| **Zambia Centre 2** | 22.4 | 0 | 0.8 | 4.5 | 77.4% | 11.3 (5.1) |
| **Zimbabwe** | 11.5 | 1 | 0.5 | 4.7 | 100.0% | 16.3(9.6) |
| **All sites** | **14.1** | **2.6** | **0.8** | **10.9** | **74.7%** | **22.75 (19.36)** |
