# Supplementary material for: Incidence of eclampsia and related complications across 10 low- and middle-resource geographical regions: Secondary analysis of a cluster randomised controlled trial
Source: PLoS Med. 2019 Mar 29;16(3):e1002775. doi: 10.1371/journal.pmed.1002775 (PMC6440614; doi:10.1371/journal.pmed.1002775)
Supplement: S2 Table — (DOCX) [file pmed.1002775.s003.docx]

**S2 Table: Eclampsia by Site and by intervention**

| **Site** |  | **Eclampsia^1^** |  |  |  |  |
| --- | --- | --- | --- | --- | --- | --- |
|  |  | Overall | Pre-intervention | Post-intervention | Unadjusted Comparison | Adjusted Comparison |
| **Ethiopia** | Rate per 10,000 deliveries  (n/N) | 57.3  203/35429 | 55.4  135/24390 | 61.6  68/11039 | 1.11 (0.83-1.49) | 1.02 (0.62-1.68) |
| **Haiti** | Rate per 10,000 deliveries  (n/N) | 83.8  125/14910 | 70.4  54/7670 | 98.1  71/7240 | 1.40 (0.98-1.99) | 0.45 (0.22-0.92) |
| **India** | Rate per 10,000 deliveries  (n/N) | 37.2  85/22876 | 52  60/11531 | 22  25/11345 | 0.42 (0.26-0.67) | 0.57 (0.23-1.39) |
| **Malawi** | Rate per 10,000 deliveries  (n/N) | 107.1  666/62165 | 93.3  450/48243 | 155  216/13922 | 1.67 (1.42-1.97) | 2.38 (1.83-3.10) |
| **Sierra Leone** | Rate per 10,000 deliveries  (n/N) | 142.0  338/23,806 | 228  48/2106 | 134  290/21700 | 0.58 (0.43-0.79) | 0.89 (0.62-1.29) |
| **Uganda Centre 2** | Rate per 10,000 deliveries  (n/N) | 27.6  167/60502 | 27.3  101/37003 | 28.1  66/23499 | 1.02 (0.76-1.40) | 3.5 (1.81-6.73) |
| **Uganda Centre 1** | Rate per 10,000 deliveries  (n/N) | 43.7  559/127817 | 37.8  94/24886 | 45.2  465/102931 | 1.19 (0.96-1.49) | 1.29 (0.96-1.73) |
| **Zambia Centre 1** | Rate per 10,000 deliveries  (n/N) | 19.6  242/123504 | 29.0  140/48252 | 13.6  102/75224 | 0.47 (0.36-0.60) | 0.52 (0.31-0.88) |
| **Zambia Centre 2** | Rate per 10,000 deliveries  (n/N) | 33.1  89/26869 | 38.4  32/8343 | 30.8  57/18526 | 0.80 (0.52-1.24) | 1.05 (0.50-2.22) |
| **Zimbabwe** | Rate per 10,000 deliveries  (n/N) | 56.8  218/38383 | 57.4  200/34814 | 50.4  18/3569 | 0.88 (0.54-1.42) | 1.18 (0.67-2.08) |
| **All sites** | Rate per 10,000 deliveries  (n/N) | **50.2**  **2692/536233** | **53.1**  **1314/247238** | **47.7**  **1378/288995** | **0.90 (0.83–0.97)** | **1.30 (0.82-2.05)** |
