# Supplementary material for: Incidence of eclampsia and related complications across 10 low- and middle-resource geographical regions: Secondary analysis of a cluster randomised controlled trial
Source: PLoS Med. 2019 Mar 29;16(3):e1002775. doi: 10.1371/journal.pmed.1002775 (PMC6440614; doi:10.1371/journal.pmed.1002775)
Supplement: S3 Table — (DOCX) [file pmed.1002775.s004.docx]

**S3 Table: Maternal death and ICU admission with HDP and stroke by site**

| **Site** |  | **Maternal Death with HDP** |  |  | **ICU admission with HDP** |  |  | **Stroke** |
| --- | --- | --- | --- | --- | --- | --- | --- | --- |
|  |  | Total | With eclampsia | Without eclampsia | Total | Eclampsia  (% Cases; n/N) | Other HDP |  |
| **Ethiopia** | Rate per 10,000 deliveries (n/N)  % Cases (n/N) | 4.8 (17/35429) | 4.0 (14/35429)  6.9% (14/203) | 0.8 (3/35429) | 11.3 (40/35429) | 7.6 (27/35429) 13.3% (27/203) | 3.7 (13/35429) | 0.6 (2/35429) |
| **Haiti** | Rate per 10,000 deliveries (n/N)  % Cases (n/N) | 12.1 (18/14910) | 12.1 (18/14910)  14.4% (18/125) | 0.0 (0/14910) | 12.1 (18/14910) | 8.1 (12/14910)  9.6% (12/125) | 0.67 (1/14910) | 3.4 (5/14910) |
| **India** | Rate per 10,000 deliveries (n/N)  % Cases (n/N) | 3.5 (8/22876) | 1.7 (4/22876)  4.7% (4/85) | 1.7 (4/22876) | 3.5 (8/22876) | 2.6 (6/22876)  7.1% (6/85) | 0.9 (2/22876) | 1.7 (4/22876) |
| **Malawi** | Rate per 10,000 deliveries (n/N)  % Cases (n/N) | 3.9 (24/62165) | 3.2 (20/62165)  3.0% (20/666) | 0.6 (4/62165) | 18.3 (114/62165) | 5.6 (35/62165)  5.3% (35/666) | 12.7 (79/62165) | 0 (0/62165) |
| **Sierra Leone** | Rate per 10,000 deliveries (n/N)  % Cases (n/N) | 19.7 (47/23806) | 15.5 (37/23806) 10.9% (37/338) | 4.2 (10/23806) | 0.0 (0/23806) | 0.0 (0/23806)  0.0% (0/338) | 0 (0/23806) | 1.3 (3/23806) |
| **Uganda Centre 1** | Rate per 10,000 deliveries (n/N)  % Cases (n/N) | 5.8 (74/127817) | 4.7 (60/127817) 10.7% (60/559) | 1.1 (14/127817) | 0.5 (6/127817) | 0.4 (5/127817)  0.9% (5/559) | 0.1 (1/127817) | 0.8 (10/127817) |
| **Uganda Centre 2** | Rate per 10,000 deliveries (n/N)  % Cases (n/N) | 2.6 (16/60502) | 2.1 (13/60502)  7.8% (13/167) | 0.5 (3/60502) | 0 (0/60502) | 0 (0/60502)  0% (0/167) | 0 (0/60502) | 0 (0/60502) |
| **Zambia Centre 1** | Rate per 10,000 deliveries (n/N)  % Cases (n/N) | 0.9 (11/123504) | 0.4 (5/123504)  2.1% (5/242) | 0.5 (6/123504) | 87.9 (1085/123504) | 19.6 (242/123504) 100% (242/242) | 68.3 (843/123504) | 0.4 (5/123504) |
| **Zambia Centre 2** | Rate per 10,000 deliveries (n/N)  % Cases (n/N) | 2.2 (6/26869) | 1.1 (3/26869)  3.4% (3/89) | 1.1 (3/26869) | 8.9 (24/26869) | 6.3 (17/26869)   19% (17/89) | 2.6 (7/26869) | 0.4 (1/26869) |
| **Zimbabwe** | Rate per 10,000 deliveries (n/N)  % Cases (n/N) | 4.2 (16/38383) | 3.1 (12/38383)  5.5% (12/218) | 1.0 (4/38383) | 8.3 (32/38383) | 6.0 (23/38383) 10.6% (23/218) | 2.3 (9/38383) | 0.8 (3/38383) |
| **All sites** | Rate per 10,000 deliveries (n/N)  % Cases (n/N) | **4.4 (237/536233)** | **3.5 (186/536233) 6.9% (186/2692)** | **1.0(51/536233)** | **24.7 (1322/536223)** | **6.8 (367/536233) 13.6% (367/2792)** | **17.8 (955/536233)** | **0.6 (33/536233)** |
