# Supplementary material for: Incidence of eclampsia and related complications across 10 low- and middle-resource geographical regions: Secondary analysis of a cluster randomised controlled trial
Source: PLoS Med. 2019 Mar 29;16(3):e1002775. doi: 10.1371/journal.pmed.1002775 (PMC6440614; doi:10.1371/journal.pmed.1002775)
Supplement: S4 Table — (DOCX) [file pmed.1002775.s005.docx]

**S4 Table: Characteristics by Site**

| **Site** |  | **Antenatal Eclampsia^1^** | **Postnatal Eclampsia** | **Age <20** | **Age 20-34** | **Age 35 or over** |
| --- | --- | --- | --- | --- | --- | --- |
| **Ethiopia** | Rate per 10,000 deliveries (n/N)  % Cases (n/N) | 52.2 (185/35429) 91.1% (185/203) | 5.1 (18/35429)  8.9% (18/203) | 5.6 (20/35429)  9.9% (20/203) | 46.6 (165/35429) 81.3% (165/203) | 5.1 (18/35429)  8.9% (18/203) |
| **Haiti** | Rate per 10,000 deliveries (n/N)  % Cases (n/N) | 78.5 (117/14910) 93.6% (117/125) | 5.4 (8/14910)  6.4% (8/125) | 24.8 (37/14910) 29.6% (37/125) | 53.0 (79/14910) 63.2% (79/125) | 6.0 (9/14910)  7.2% (9/125) |
| **India** | Rate per 10,000 deliveries (n/N)  % Cases (n/N) | 33.2 (76/22876) 89.4% (76/85) | 3.9 (9/22876)  10.6% (9/85) | 5.7 (13/22876)  15.3% (13/85) | 31.0 (71/22876) 83.5% (71/85) | 0.4 (1/22876)  1.2% (1/85) |
| **Malawi** | Rate per 10,000 deliveries (n/N)  % Cases (n/N) | 98.1 (610/62165) 91.6% (610/666) | 9.0 (56/62165)  8.4% (56/666) | 54.2 (337/62165) 50.1% (337/666) | 46.5 (289/62165) 43.4% (289/666) | 6.4 (40/62165)  6.0% (40/666) |
| **Sierra Leone** | Rate per 10,000 deliveries (n/N)  % Cases (n/N) | 135.2 (322/23806)  95.3% (322/338) | 6.7 (16/23806)  4.7% (16/338) | 48.3 (115/23806) 34.0% (115/338) | 86.1 (205/23806) 60.1% (205/338) | 7.6 (18/23806)  5.3% (18/338) |
| **Uganda Centre 1** | Rate per 10,000 deliveries (n/N)  % Cases (n/N) | 38.9 (497/127817) 88.9% (497/559) | 4.9 (62/127817) 11.1% (62/559) | 10.6 (135/127817) 24.2% (135/559) | 30.8 (394/127817) 70.5% (394/559) | 2.3 (30/127817)  5.4% (30/559) |
| **Uganda Centre 2** | Rate per 10,000 deliveries (n/N)  % Cases (n/N) | 27.1 (164/60502) 98.2% (164/167) | 0.5 (3/60502)  1.8% (3/167) | 8.8 (53/60502)  31.7% (53/167) | 16.0 (97/60502) 58.1% (97/167) | 2.8 (17/60502)  10.2% (17/167) |
| **Zambia Centre 1** | Rate per 10,000 deliveries (n/N)  % Cases (n/N) | 18.9 (234/123504) 96.7% (234/242) | 0.6 (8/123504)  3.3% (8/242) | 6.2 (77/123504) 31.8% (77/242) | 11.7 (144/123504) 59.5% (144/242) | 1.7 (21/123504)  8.7% (21/242) |
| **Zambia Centre 2** | Rate per 10,000 deliveries (n/N)  % Cases (n/N) | 30.9 (83/26869) 93.3% (83/89) | 22.3 (6/26869)  6.7% (6/89) | 10.8 (29/26869) 32.6% (29/89) | 17.9 (48/26869) 53.9% (48/89) | 4.5 (12/26869)  13.5% (12/89) |
| **Zimbabwe** | Rate per 10,000 deliveries (n/N)  % Cases (n/N) | 53.9 (207/38383) 95.0% (207/218) | 2.9 (11/38383)  5.0% (11/218) | 20.3 (78/38383) 35.8% (78/218) | 32.3 (124/38383) 56.9% (124/218) | 4.2 (16/38383)  7.3% (16/218) |
| **All sites** | **Rate per 10,000 deliveries (n/N)**  **% Cases (n/N)** | **46.5 (2495/536233) 92.7% (2495/2692)** | **3.7 (197/536233) 7.3% (197/2692)** | **16.7 (894/536233) 33.2% (894/2692)** | **30.1 (1616/536233) 60.0% (1616/2692)** | **3.4 (182/536233) 6.8% (182/2692)** |

^1.^Includes eclampsia on day of delivery.
