# Supplementary material for: Incidence of eclampsia and related complications across 10 low- and middle-resource geographical regions: Secondary analysis of a cluster randomised controlled trial
Source: PLoS Med. 2019 Mar 29;16(3):e1002775. doi: 10.1371/journal.pmed.1002775 (PMC6440614; doi:10.1371/journal.pmed.1002775)
Supplement: S5 Table — (DOCX) [file pmed.1002775.s006.docx]

**S5 Table: Perinatal outcomes for mothers with HDP by Site**

| **Sites** |  | **All HDP** | **HDP with Antenatal eclampsia** | | | **HDP causing ICU admission or maternal death without eclampsia** | | |
| --- | --- | --- | --- | --- | --- | --- | --- | --- |
|  |  | **All stillbirth and neonatal death in women with HDP** | **All still births and neonatal deaths** | **Pregnancies with Stillbirth** | **Pregnancies Neonatal Death^1^** | **All still births and neonatal deaths** | **Pregnancies with Stillbirth** | **Pregnancies with Neonatal Death^1^** |
| **Ethiopia** | n/N (%) | **42/100 (42%)** | **36/185 (19.4%)** | 32/185 (17.3%) | 4/185 (2.2%) | **6/15 (40.0%)** | 6/15 (40.0%) | 0/15 (0%) |
| **Haiti** | n/N (%) | **17/118 (14.4%)** | **17/117 (14.5%)** | 17/117 (14.5%) | 0/117 (0%) | **0/1 (0%)** | 0/1 (0%) | 0/1 (0%) |
| **Sierra Leone** | n/N (%) | **78/334 (37.8%)** | **72/322 (22.4%** | 59/322 (18.3%) | 13/322 (40.3%) | **6/12 (50%)** | 5/12 (41.7%) | 1/12 (8.3%) |
| **India** | n/N (%) | **13/81 (16.0%)** | **10/76 (13.2%)** | 3/76 (3.9%) | 7/76 (9.2%) | **3/5 (60%)** | 2/5 (40.0%) | 1/5 (20.0%) |
| **Malawi** | n/N (%) | **35/691 (5.1%)** | **25/610 (41.0%)** | 20/610 (3.3%) | 5/610 (0.8%) | **10/81 (12.3%)** | 9/81 (11.1%) | 1/81 (1.2%) |
| **Uganda Centre 1** | n/N (%) | **122/514 (23.7%)** | **115/497 (23.1%)** | 91/497 (18.3%) | 24/497 (4.8%) | **7/17 (41.2%)** | 6/17 (35.3%) | 1/17 (5.9%) |
| **Uganda Centre 2** | n/N (%) | **16/167 (9.6%)** | **14/164 (8.5%)** | 14/164 (8.5%) | 0/164 (0%) | **2/3 (66.7%)** | 2/3 (66.7%) | 0/3 (0%) |
| **Zambia Centre 1** | n/N (%) | **237/1077 (22.0%)** | **50/234 (21.4%)** | 44/234 (18.8%) | 6/234 (2.6%) | **187/843 (22.2%)** | 162 / 843 (19.2%) | 25/846 (3.0%) |
| **Zambia Centre 2** | n/N (%) | **15/93 (16.1%)** | **11/83 (13.3%)** | 7/83 (8.4%) | 4/83 (4.8%) | **4/10 (40%)** | 3/10 (33.3%) | 1/10 (10.0%) |
| **Zimbabwe** | n/N (%) | **50/218 (22.9%)** | **47/207 (22.7%)** | 35/207 (16.9%) | 12/207 (58.0%) | **3/11 (27.3%)** | 2/11 (18.2%) | 1/11 (9.1%) |
| **All sites** | **n/N (%)** | **625/3493 (17.9%)** | **397/2495 (15.9%)** | **322/2495 (12.9%)** | **75/2495 (3.0%)** | **228/998 (22.8%)** | **197/998 (19.7%)** | **31/998 (3.1%)** |
